# Introducing Telepsychiatry to Medical Students with Simulated Patients: An Innovation by Necessity

**DOI:** 10.15694/mep.2020.000187.1

**Published:** 2020-09-03

**Authors:** Michael Miller, Dawnelle Schatte, Karen Szauter

**Affiliations:** 1 University of Texas Medical Branch

**Keywords:** clinical skills, medical education, remote education, telemedicine, telepsychiatry, e-learning, standardized patient

## Abstract

This article was migrated. The article was marked as recommended.

**Introduction**: The abrupt discontinuation of medical student-patient contact due to the covid-19 pandemic resulted in a rapid change to virtual teaching. Student education was restructured to include online cases, small group discussions, synchronous and asynchronous lectures, and modified problem-based learning and team-based learning sessions. However, the key focus of the clerkship experience, contact with patients, was missing.

**Process**: The Psychiatry Clerkship directors have previously provided complex simulated encounters to students using video-taped encounters of physician-simulated patient interactions to teach and assess student note writing skills. This concept was adapted to a live encounter for individual students on the psychiatry clerkship. Students reviewed the patient chart, performed the encounter, provided an oral patient presentation to faculty, and wrote a patient note. Individualized feedback was provided for each step of the process.

**Outcomes**: The process was well received by students and faculty and provided an opportunity to directly observe student skills despite distancing from direct patient care. The simulated patients had a very positive experience and appreciated the opportunity to advance their own skills while contributing to student’s education.

**Discussion**: Removing students from clinical sites stimulated the rapid development of a process to observe learners involved in patient encounters. These educational sessions allowed direct observation of skills required in the initial evaluation of a patient presenting to psychiatry for care.

## Introduction

The first two years of our medical school curricula focus on foundational science concepts. The curriculum uses problem-based learning as the primary educational method, with hands-on laboratory sessions and limited classroom didactics. Running in parallel is the Practice of Medicine, two year-long courses focused on learning the techniques needed for patient interactions including the medical interview, physical examination, oral case presentation, and documentation of patient information. When students enter the clinical clerkship phase of training the learning focus rapidly changes from small-group sessions to learning during direct patient care with the application of medical knowledge and clinical skills.

The recent pandemic caused an abrupt cessation of student-patient contact. Following national guidelines, all students were removed from clinical rotations and required to shelter at home. Clerkship directors from all disciplines were left with the challenge of maintaining some level of clinical education remotely. Commercial companies generously offered online cases for reduced or no cost, and educators reached out with personally developed learning programs in their respective disciplines. However, the key part of the clerkship year, direct patient contact, remained a challenge.

Our psychiatry clerkship directors have long recognized the challenges of teaching students to effectively work with patient with mental health concerns. In addition to guiding students through the unique features of the psychiatric interview, educators work to facilitate the skills needed to recognize patient’s behavioral cues and provide a supportive environment for the patient to express him or herself. Students learn through direct observation of faculty or residents interacting with patients and their own patient encounters. Observation and assessment of student-patient interactions is fundamental to determining students’ strengths and areas to focus learning. This prompted the development of a multi-step remote student-patient encounter.

## Educational Innovation

As a discipline, psychiatry is uniquely suited to telemedicine encounters because of limited physical contact with patients. Telepsychiatry has been slow to become a standard of practice due to issues with technology, reimbursement, and training specific to working with patients remotely ( Lauckner and Whitten, 2016; Cowan *et al.,* 2019). With limitations of face to face contact created by the pandemic, patient care via telephone or video encounters has become a new practice standard. We modeled this student learning opportunity by considering each step of a new patient visit and provided some simplified guidelines for telehealth encounters to the students in preparation for the encounter.

To create a safe learning environment for all involved, simulated patients (SPs) were recruited for this encounter. The application of SP methodology in psychiatry teaching and assessment is well established and has been used at our institution previously ( Shirazi *et al.,* 2014). A new scenario was developed for this session involving a mature adult woman with a mood disorder. The challenge was felt to be core to work in psychiatry and appropriate for the level of learners. Two experienced SPs were recruited and trained to the scenario remotely. The Zoom ^TM^ platform, selected by our institution for remote education during the pandemic, was used for training. This allowed the SPs to train to the scenario together, calibrating case details and affect, and gave the SPs an opportunity to trouble shoot issues with the computer system. Both SPs were expected to be present for all student encounters and alternated in their role as observer vs patient. This provided instant patient back up if internet connections or other technological challenges precluded the SP from being able to join by video when cued.

The encounter was framed for the student as a new patient referral to psychiatry by her primary care provider. To detail the patient background a chart was developed using a feature of our institutional electronic medical record (EMR), Epic TrainPlay - a non-production environment, describing recent clinical visits, vitals, medications, and reason for referral. Students were instructed to review the EHR prior to the patient encounter. Each student was given an appointment time for the patient visit and the link to log into Zoom ^TM^. The waiting room feature was used and allowing faculty to admit the student to the Zoom ^TM^ session at the designated time. The session began with faculty and student on video, allowing sound and screen issues to be checked, and giving an opportunity for the student to address any logistical details. When the student confirmed readiness to begin, faculty disabled his/her video and the SP enabled hers. Students were allotted 25 minutes for direct patient interaction. Faculty remained in the session watching the encounter but remained off video, speaking only to remind the students that the time for the encounter was ending. At the conclusion of the patient encounter the students was given a fifteen-minute break, then logged into another Zoom ^TM^ meeting where a faculty member was waiting to hear the oral presentation of the patient encounter. After providing the oral presentation, the student was instructed to document the encounter in EPIC as would have been done with a routine encounter in the clinic.

Student skills were assessed by both faculty and the SPs. Faculty provided written feedback to the student regarding the patient encounter, and the SPs evaluated student interpersonal skills using a modified version of the CARE form ( Mercer *et al.,* 2004). The oral presentation and patient note were also scored, and feedback was provided to the student. Oral presentations were rated based on details of the patient history, mental status exam findings, problem representation statement, differential diagnosis and rationale, along with plan for next course of action. Documentation in the EMR was graded based on a standardized rubric previously used in the psychiatry clerkship. Expectations for inclusion included details of the history, mental status exam, initial assessment with differential diagnosis and details of recommended steps in management. Students received written feedback on both post-patient encounter exercises, with specific notations on recommended areas for improvement.

## Outcomes

Individual sessions have been administered to two cohorts (n=58) of students during the pandemic. Informal feedback from faculty, students, and standardized patients about the experience was overall positive.

Students appreciated the opportunity to engage in patient contact in this unique setting. Faculty noted that watching individual student interviews was helpful in determining specific problems in the approach to a patient presenting for a mental health visit. To better explore these problems, a modified version of the oral presentation faculty grading form, featuring expanded scoring scales and more weight toward clinical reasoning, was developed and used in parallel with the original form on the most second cohort of students. The modified form grades were similar but more specific, showing potential for further use and development to reflect student performance more accurately and provide meaningful feedback.

Areas for further development include obtaining formal feedback from students and standardized patients to inform future modifications of the process.

## Discussion

Our current medical school curriculum has been notably impacted by the pandemic restrictions. Although the clerkships have moved to virtual curricula to keep students actively engaged in their educational program, modified clerkship clinical times will follow as soon as patient contact restrictions are modified. This exercise has challenged students to work through a patient encounter in psychiatry and has allowed identification of students who were struggling with various steps in a patient care scenario. The multi-step assessment had allowed us to provide guidance on how to improve with the foreshorten time of face-to-face curriculum.

This activity provides a rich opportunity to observe students in a remote setting. Although the introduction of this process was necessitated by the loss of student-patient contact, we plan to continue using this program for teaching in the future.

## Take Home Messages

• COVID-19 institutional restrictions challenge medical school clinical clerkships to meet objectives for student’s learning, particularly patient encounters with interviewing and examination skills.
• To meet these criteria, we have created a virtual psychiatry telehealth visit with standardized patients and rapid feedback for professional growth.
• This virtual encounter could be used by clerkships in psychiatry and other specialties and fields to educate and evaluate clinical students in clinical skills areas while in-person interactions are not an option, and beyond.

## Notes On Contributors

Dr. Michael Miller is an assistant professor of psychiatry and co-director of the third year medical student clerkship at the University of Texas Medical Branch. ORCID: https://orcid.org/0000-0003-1054-8216

Dr. Dawnelle Schatte is an associate professor of psychiatry and director of the third year medical student clerkship at the University of Texas Medical Branch. ORCID: https://orcid.org/0000-0002-4191-5579

Dr. Karen Szauter is assistant dean, educational affairs, professor of internal medicine, and co-director of the third year medical student clerkship at the University of Texas Medical Branch. ORCID: https://orcid.org/0000-0002-2064-3535

## Declarations

The author has declared that there are no conflicts of interest.

## Ethics Statement

This is a description of a curricular innovation and includes no data describing human subjects. This work focused on a new educational process and was not set up as research or analyzed with student data. Per the University of Texas Medical Branch Institutional Review Board, the project did not meet the definition of human subject research at 45 CFR 46.102 as it did not yield generalizable knowledge and was limited to experiences at a single institution. Therefore, IRB approval or oversight was not required. A letter obtained from our IRB is available upon request.

## External Funding

This article has not had any External Funding
