# Supplementary material for: Introducing Telepsychiatry to Medical Students with Simulated Patients: An Innovation by Necessity
Source: MedEdPublish (2016). 2020 Sep 3;9:187. [Version 1] doi: 10.15694/mep.2020.000187.1 (PMC10699376; doi:10.15694/mep.2020.000187.1)
Supplement: Peer review history

# Reviewer report: Reviewer response for version 1

**DOI:** 10.21956/mep.20108.r31001

**Published:** 2020-09-20

**Authors:** Subha Ramani

**Affiliations:** 1 Harvard Medical School, Brigham and Women's Hospital

This review has been migrated. The reviewer awarded 3 stars out of 5 The authors have described a Telehealth patient curriculum using standardized patients to train students during their psychiatry rotation. This switch to virtual patient care was necessitated by the pandemic.They have described the intervention well trying to mimic a real patient virtual visit. The evaluation of the program would be important as they continue the curriculum. I would have liked a little more reporting of the informal opinions about this intervention.By now, there are many reports of Telehealth and a few more references would also be helpful.Lastly, this can easily be translated to real patient encounters.For all educators intending to design a Telehealth curriculum in psychiatry, this paper would be useful.

Reviewer Expertise:

NA

No decision status is available

# Reviewer report: Reviewer response for version 1

**DOI:** 10.21956/mep.20108.r31002

**Published:** 2020-09-11

**Authors:** Ken Masters

**Affiliations:** 1 Sultan Qaboos University

This review has been migrated. The reviewer awarded 3 stars out of 5 The paper details the switch to telepsychiatry in undergraduate medical training because of the pandemic. The rationale and the process has been well-documented.Although this is billed as a new intervention, and therefore the evaluation is not expected to be as rigorous as one would expect in a full research paper, I would like to see more details (and more formally presented) of the results. In addition, unless there are copyright or similar restrictions, it would be useful to see the old and the new grading forms, so that the reader can more accurately gauge the extent to which the intervention has impacted upon the process.

Reviewer Expertise:

NA

No decision status is available

# Reviewer report: Reviewer response for version 1

**DOI:** 10.21956/mep.20108.r31000

**Published:** 2020-09-10

**Authors:** Adel Altawaty

**Affiliations:** 1 Libyan International Medical University

This review has been migrated. The reviewer awarded 4 stars out of 5 This is a practical and serious attempt to go around the challenge of implementing clinical clerkship in the era of Covid-19. The article describes in detail the steps of the procedure which helps reproducing it. I think the next step would be using “suitable” real patients because this can give more natural dimension to this innovative educational approach.

Reviewer Expertise:

NA

No decision status is available

# Reviewer report: Reviewer response for version 1

**DOI:** 10.21956/mep.20108.r30999

**Published:** 2020-09-05

**Authors:** P Ravi Shankar

**Affiliations:** 1 American International Medical University

This review has been migrated. The reviewer awarded 4 stars out of 5 This is an interesting article about telepsychiatry and virtual student interaction with simulated patients during the clerkship. The authors have described the planning and the implementation process in detail. Virtual encounters will continue to have an important role even after in person consultations in the physical space are allowed after the pandemic. They can supplement and strengthen in person learning. There may be challenges in interacting with ‘real’ psychiatric patients. How did the authors ensure that the students were introduced to these challenges?

Reviewer Expertise:

NA

No decision status is available
